# Reduced CCR6^+^IL-17A^+^Treg Cells in Blood and CCR6-Dependent Accumulation of IL-17A^+^Treg Cells in Lungs of Patients With Allergic Asthma

**DOI:** 10.3389/fimmu.2021.710750

**Published:** 2021-08-23

**Authors:** Xiaokun Shen, Huiyun Zhang, Hua Xie, Liping Chen, Shinan Li, Junjuan Zheng, Ruonan Chai, Zhao Wang, Yanyan Zang, Shaoheng He

**Affiliations:** ^1^Institute of Translation Medicine, First Affiliated Hospital of Jinzhou Medical University, Jinzhou, China; ^2^Institute of Translation Medicine, Shenyang Medical College, Shenyang, China; ^3^People’s Liberation Army (PLA) Center of Respiratory and Allergic Disease Diagnosing Management, General Hospital of Shenyang Military Area Command, Shenyang, China; ^4^Respiratory Medicine Department, Second Affiliated Hospital of Shenyang Medical College, Shenyang, China; ^5^State Key Laboratory of Genetic Resources and Evolution/Key Laboratory of Healthy Aging Research of Yunnan Province, Kunming Institute of Zoology, Chinese Academy of Sciences, Kunming, China

**Keywords:** asthma, HDM, CCR6^+^ Treg, IL-17, CCL20

## Abstract

Human regulatory T (Treg) cells play a central role in controlling allergic inflammation in the airways. A reduced number of peripheral Treg cells and decreased suppressive function have been previously reported in the pathogenesis of allergic asthma. However, the characteristic role of specific Treg cell subsets and their mechanisms in the pathogenesis of allergic asthma remain unclear. In this study, we examined the proportion of different Treg cell subsets in both healthy subjects and patients with allergic asthma using flow cytometry and single-cell RNA sequencing. The migration function of the cells was compared using cell sorting and Transwell experiments. Furthermore, two allergen-challenged mouse models and a cell transfer experiment were used to examine the role of these Treg subsets. We found that the proportion of CD25^+^Foxp3^+^CD127^-^ Treg cells in the peripheral blood of patients with allergic asthma was lower than in those of healthy subjects. Furthermore, the circulating Treg cells expressed lower levels of CCR6 and IL-17 compared with healthy subjects. The chemokine from the airway mucosa, CCL20, was abundantly expressed, and Transwell experiments further proved that this chemokine promoted CCR6^+^ Treg cell migration *in vitro*. A mouse model induced by house dust mite (HDM) revealed that the number of CCR6^+^ Treg cells in the lung tissue increased remarkably. The incidence of allergic asthma may be related to an increase in Treg cells secreting IL-17 in the lung tissue. Recruited CCR6^+^ Treg cells are likely to differentiate into Th17-like cells under the Th17 environment present in the lungs. IL-17 derived from Th17-like cells could be associated with the pathology of allergic asthma by promoting Th17 responses, thereby favoring HDM-induced asthma exacerbations.

## Introduction

Asthma is a heterogeneous disease that affects approximately 300 million people worldwide (1, 2). The inflammatory response to common environmental allergens during allergy and asthma has been extensively studied in the past decades (3). For example, the involvement of regulatory T (Treg) cells in allergic asthma has been widely reported. Treg cells, characterized by the production of anti-inflammatory cytokines, such as IL-10 and TGF-β, are mainly involved in the maintenance of tolerance (4). They can inhibit type 2 immune cells, such as T helper 2 (Th2) cells (5) and type 2 innate lymphoid cells (ILC2s) (6), as well as induce the generation of tolerogenic dendritic cells (DCs) (7). A deficiency in the count and activation of Treg cells was found during allergen exposure and exacerbation of severe asthma (8).

Our own and other research groups previously reported that Treg cells can be classified into natural Treg cells (nTreg cells), inducible Treg cells (iTreg cells), inducible costimulator (ICOS^+^ Treg cells), IL-10 producing type 1 Treg cells (Tr1 cells), CD8^+^ Treg cells, and IL-17-producing Treg cells (5, 9). Among them, IL-17A-producing Treg cells, which are only found in peripheral blood and lymphoid tissue (10), express the Th17-specific transcription factor RORγt and can be transformed from conventional Treg cells following stimulation by IL-6, IL-21, IL-23, and IL-1β (11). IL-17 secreting Treg cells also have been reported to be associated with allergic rhinitis (12) and inflammatory bowel disease (13).

CCR6 (CD196) is a G protein-coupled chemokine receptor expressed on several types of immune cells, including Th17 and Treg cells (14–16). CCR6 controls the migration of ILC2s and γδT cells into the lungs and inflamed tissues under physiological and inflammatory conditions (17, 18). The macrophage inflammatory protein-3 alpha (MIP-3α), also called CCL20, is a unique high-affinity chemokine ligand for CCR6 (19), which promotes the development of Th2 immune responses and allergic inflammation by recruiting memory dendritic cells (mDCs) and memory CCR6^+^CD4^+^ T cells to the lungs (20, 21). Elevated levels of IL-17 are associated with inflammatory diseases, such as rheumatoid arthritis and asthma (22), as well as inflammatory cells, particularly neutrophil recruitment into airways (23, 24). Because IL-17-positive cells were found in bronchial biopsies from severe asthmatics (25) and pathogenic conversion of Foxp3^+^ T cells into Th17 cells are involved in inflammatory diseases (26, 27), we anticipated that IL-17-producing Treg cells might participate in the development of airway inflammation.

The present study investigated the role of Treg cells subtypes and CCL20-CCR6-IL-17A-related mechanism in the development of allergic asthma.

## Materials and Methods

### Ethics Statement

Our study was conducted in accordance with the guidelines of the Declaration of Helsinki and the Principles of Good Clinical Practice. All patients and healthy controls who participated in this study provided written informed consent, and our research was approved by the ethics committee of the First Affiliated Hospital of Jinzhou Medical University, China. The clinical trial registration number is ChiCTR-BOC-16010279, and the URL is http://www.chictr.org.cn/. The registration date was December 28th 2016.

### Healthy Controls and Patients With Allergic or Non-Allergic Asthma

Seventeen healthy controls (HC) with no history of allergy or asthma, 42 allergic asthmatics (aAS), and five non-allergic asthmatics (naAS) were recruited for this study. Allergic asthma was diagnosed according to the criteria of the Global Initiative for Asthma (GINA reports). In brief, aAS subjects were diagnosed based on physical examination, history, serum IgE levels, and skin test responses to allergens. And naAS subjects were diagnosed with asthma who had the negativity of 14 skin prick tests to common environmental allergens(such as Artemisia, house dust mite, Platanus pollen, cat fur, dog fur and so on). HC were defined as nonsmoking, negative skin test results and no evidence of lung disease. HC were recruited from the first affiliated hospital of Jinzhou medical university. Patients with asthma were recruited from the general hospital of Shenyang military area command and the second affiliated hospital of Shenyang medical college. Peripheral blood samples were collected from all individuals. The clinical characteristics of enrolled subjects are listed in **Table 1**.

**Table 1:** Clinical characteristics of healthy controls and patients with asthma.

	HC (N = 17)	aAS (N = 42)	nAS (N = 5)
**Age, year**	(40 ± 2.3)	(39 ± 2.9)	(41 ± 0.3)
**Gender (Male/Female)**	8/9	16/26	1/4
**History, year**	NA	4.2 ± 0.8	4.1 ± 1.2
**Artemisia (+)**	0	8	0
**House dust mite (+)**	0	27	0
**Platanus pollen (+)**	0	7	0
**Cat fur (+)**	0	5	0
**Dog fur (+)**	0	3	0
**Glucocorticoid treatment (Before)**	0	9	0
**Glucocorticoid treatment (within one month)**	0	0	0

The mean ± SEM are shown. Specific allergens were examined using a skin prick test. HC, healthy control; NA, not applicable; aAS, allergic asthmatics; naAS, non-allergic asthmatics.

### Isolation of PBMCs

Fresh peripheral blood mononuclear cells (PBMCs) were isolated by density gradient centrifugation using Ficoll-Paque Plus (GE Healthcare). PBMCs were then washed twice with 1_x_ phosphate buffered saline (PBS) and resuspended in RPMI 1640 medium (Gibco, Grand Island, NY, USA) supplemented with 10% heat-inactivated foetal bovine serum (HyClone, Logan, UT, USA) and 100 units/mL penicillin/streptomycin.

### Flow Cytometry

PBMCs (1 × 10^6^ cells) were cultured for five hours with PMA (50 ng/mL; Sigma Chemical, Saint-Louis, MO, USA); Ionomycin (1 μg/mL; Euromedex) and Brefeldin A (10 μM; BD Bioscience) were added during the last 1 h of culture. Cells were harvested and stained with Panel A antibodies: CD3-Percp-Cy5.5, CD8-FITC, CD25-PE, and CD127-Brilliant Violet 510; Panel B antibodies: CD3-Percp-Cy5.5, CD8-FITC, CCR6-PE, CD278-PE/Cy7, and CD25-Brilliant Violet 510. After staining of cell-surface markers, cells were fixed and permeabilized with the Foxp3 Fixation/Permeabilization Buffer (BioLegend) according to the manufacturer’s protocols. Subsequently, the cells were stained with Panel A: IL-10-PE/Cy7, TGF-β-Brilliant Violet 421, and Foxp3-Alexa-Fluor 647; Panel B: Foxp3-Alexa-Fluor 647, and IL17A-Brilliant Violet 421. All antibodies were purchased from BioLegend (San Diego, CA, USA). The detailed antibodies information were listed in **Table 2**. In humans, Treg cells are classically defined as CD4^+^CD25^+^Foxp3^+^ T cells. In this study, Treg cells were first defined as single cells within the lymphocyte gate. Second, these cells were CD3^+^CD25^+^Foxp3^+^ yet Zombie^−^ and CD8^−^ to exclude dead cells and CD8^+^ Treg cells, respectively (**Supplementary Figure 1**).

**Table 2:** Fluorochrome-labelled antibodies used in flow cytometry analyses.

Antibodies (anti-human)	Fluorochrome	Company	Clone
CD3	PercP-Cy5.5	Biolegend	HIT3a
CD4	PE	BD	RPA-T4
CD4	APC/Cyanine7	Biolegend	OKT4
CD8a	FITC	Biolegend	HIT8a
CD25	PE	Biolegend	BC96
CD25	Brilliant Violet 510	Biolegend	M-A251
CD127	Brilliant Violet 510	Biolegend	A019D5
Foxp3	Alexa-Fluor647	Biolegend	206D
CCR6	PE	Biolegend	G034E3
IL-17A	Brilliant Violet 421	Biolegend	BL168
IL-10	PE/Cy7	Biolegend	JES3-9D7
TGF-β	Brilliant Violet 421	Biolegend	TW4-2F8
CD278(ICOS)	PE/Cy7	Biolegend	C398.4A
**Antibodies (anti-mouse)**	**Fluorochrome**	**Company**	**Clone**
Foxp3	PercP-Cy5.5	BD	R16-715
CD4	PE	Biolegend	GK1.5
CD127	Brilliant Violet 510	Biolegend	ATR37
IL-17A	Brilliant Violet 421	Biolegend	TC11-18H10.1
CD25	APC/Cyanine7	Biolegend	PC61
CCR6	Alexa-Fluor647	Biolegend	29-2L17
Ly-6G/Ly-6C	FITC	Biolegend	RB6-8C5
CD11b	APC	Biolegend	M1/70

### Treg Cells Sorting

Fresh human PBMCs were stained with monoclonal antibodies, such as PercP-Cy5.5-anti-human CD3, APC/Cy7-anti-human CD4, PE-anti-human CD25, and Brilliant Violet 510-anti-human CD127 (all purchased from BioLegend, San Diego, CA, USA), in 0.5% BSA + 2 mM EDTA in PBS. Splenocytes were obtained from the spleens of mice by mechanical disruption and through a 200-gauge steel mesh. Splenocytes were lysed with 1_X_ red blood cells (RBCs) lysis buffer to remove RBCs. First, magnetic microbeads (Miltenyi Biotec) bead-based enrichment of CD4^+^T cells was performed. Then, cells were stained with monoclonal antibodies, such as PE-anti-mice CD4, APC/Cyanine7-anti-mice CD25, and Alexa-Fluor647-anti-mice CCR6 (all purchased from BioLegend), in 0.5% BSA + 2 mM EDTA in PBS. Human Treg cells and murine Treg cells were sorted using a SH800S cell sorter (SONY, Japan) and analyzed with the SH800 software (SONY, Japan). Human Treg cells were gated as CD3^+^CD4^+^CD25^+^CD127^-^ within the lymphocyte gate, which excluded cell debris, doublets, and dead cells (**Supplementary Figure 2**). Mice Treg cells were gated as CD4^+^CD25^+^ within the lymphocyte gate which excluded cell debris, doublets, and dead cells (**Supplementary Figure 2**). The purity of the sorted cell population was above 95% for human and 91% for mice, which was verified using post-sorting flow cytometry.

### Induced Sputum, Chemokine, and Cytokine Analysis

Sputum was induced with hypertonic saline (4.5%) and processed as previously described (28). The amount of CCL20 of induced sputum was determined using the human CCL20/MIP-3 alpha Quantikine ELISA Kit (R&D Systems), and the mouse MIP-3 alpha/CCL20 AccuSignal ELISA Kit (Rockland) was used for the analysis of mice CCL20. The levels of IL-17A, IL-4, and IgE were determined using the ELISA kits from Dakewe Biotech (Shenzhen, China).

### Transwell Assays

To assess the migratory properties, Treg cells (1 × 10^5^) were seeded in the upper chamber of a 5-μm 24-well transwell plate (Corning Costar, Corning, NY) in 100 μL of RPMI 1640 medium containing 10% FBS. A gradient of 100 ng/mL recombinant chemokines CCL20 (PeproTech, Germany) was added to the lower compartment. Migration was assayed for 2.5 h at 37 °C, 5% CO_2_, and subsequently the inserts were removed. Migrated Treg cells were enumerated with an automatic cell counter (Countstar BioMed, ALIT Life Science, Shanghai, China).

### Detection of *CD4, Foxp3, CD25, CD8A*, and *CD8B* Expression in Treg Cells Using Whole-Transcriptome ScRNA-seq Data

Expression of *CD4*, *Foxp3*, *CD25*, *CD8A*, and *CD8B* was assessed in our 10X Genomics datasets combining 3’-mRNA and surface protein expression. Foxp3^hi^ cells were defined as cells expressing one or more copies of Foxp3. More than one thousand Foxp3^hi^ cells obtained from two HC and two patients with allergic asthma were analyzed in this study. The sequencing depth was 20000 to 50000 reads per cell or nuclei. We used the Seurat package to perform ScRNA-seq analysis. The function of UMAPplot, RidgePlot, and FeatureScatter were used to plot the figures. The detailed procedure on single-cell RNA-seq and ScRNA-seq data processing is provided in the **Supplementary Material**.

### Animal Study

C57BL/6 mice (6–8 weeks old) were purchased from Beijing Vital River Laboratory Animal Technology Co., Ltd. (Beijing, China). eGFP transgenic mice and CCR6^-/-^ mice were obtained from the Model Animal Research Center (Nanjing, China), provided by the Jackson Laboratory. All mice were raised under specific pathogen-free conditions. The Committee on Animal Experimentation of Jinzhou Medical University approved the animal procedures used in this study.

### Induced Allergic Airway Inflammation

For ovalbumin (OVA)-induced airway inflammation, mice were sensitized by intraperitoneal injection with 50 mg of OVA in 1 mg of Alhydrogel (CSL), and after one week, they were challenged with 50 mg of OVA intranasally (i.n.) for seven days. Non-sensitized mice received 1 mg of Alhydrogel in 0.9% saline and were challenged with sterile saline. Mice were harvested and examined on Day 19. For house dust mite (HDM)-induced airway inflammation, mice were sensitized and challenged with intratracheal administration of HDM extracts (Huipuyuan, Liaoning, China) with 50 μg HDM in 25 μL of 0.9% saline twice at seven day intervals. Seven days after the last sensitization, mice were challenged with 5 μg of HDM for four consecutive days, and 24 h after the last HDM challenge, mice were harvested and analyzed. Their blood was collected, after which the mice were tracheotomized. Bronchoalveolar lavage fluid (BALF) was collected for analysis using FACS, ELISA, and inflammation cell counting. To obtain cells in the bronchoalveolar lavage fluid (BALF), we performed bronchoalveolar lavage as described previously (29) with slight modifications. The whole lung was perfused through the trachea three times with 0.5 ml sterile phosphate buffered saline (PBS). After centrifugation of the BALF, cell pellets were harvested for FACS. To obtain cells in whole blood, the red blood cells (RBCs) were removed by RBC lysis buffer (BioLegend). The harvested cells were washed with 1_x_ PBS and collected for further experiments. Serum was collected, and total IgE were measured by ELISA.

### Treg Cells Identified in Mice

For surface staining, single cells were incubated with antibodies, such as anti-mice CD4 and anti-mice CD25 for 30 min at 4°C after blocking the Fc receptor, then washed twice with 1_x_ phosphate buffered saline (PBS). After staining of cell-surface markers, cells were fixed and permeabilized with a Foxp3 Staining Buffer Set (BioLegend) according to the manufacturer’s protocol and then stained with anti-mouse IL-17 and anti-mouse Foxp3 or isotype control for 1 h at 4°C. Samples were analyzed by the BD verse system (BD Biosciences). In mice, Treg cells were defined as CD4^+^CD25^+^Foxp3^+^ within the lymphocytes gate.

### Histology

Mouse lung tissues without lavage were fixed in 10% formalin and subsequently embedded in paraffin. The lung sections were stained with hematoxylin and eosin (H&E). Histopathology images were acquired on a Leica microscope (DM4000 B LED) using magnification (200×) by a DFC450C camera and Leica application suite V4.12 software. More than six fields from left and right of the lung samples were selected randomly to determine inflammatory injury, and the researcher was blinded to the treatments. Histological scores were assessed as described previously (30). Each set of sections was given a score of 0–5 for inflammation (0, no inflammation; 1, few inflammatory cells; 2, a ring of inflammatory cells one cell layer deep; 3, a ring of inflammatory cells two to four cells deep; and 4, a ring of inflammatory cells of more than four cells deep).

### Data Analysis

All flow cytometry data were analyzed using FlowJo X 10.0.7r2 (Tree Star). Statistical analysis was performed using Prism 6.0 (GraphPad Software). Pearson’s correlations were calculated using logarithmically transformed data. Comparisons of continuous variables between groups were conducted using unpaired *t-*tests (two-tailed), one-way and two-way analysis of variance, and linear regression according to the type of experiment. The *P* values of <0.05 were considered significant (*P < 0.05, **P < 0.01, ***P < 0.001 and ****P < 0.0001). Data were summarized as the mean ± SEM, unless otherwise indicated.

## Results

### The Ratio of Peripheral CCR6^+^CD25^+^Foxp3^+^ Treg Cells Decreased in Patients With Allergic Asthma

**Figure 1:**
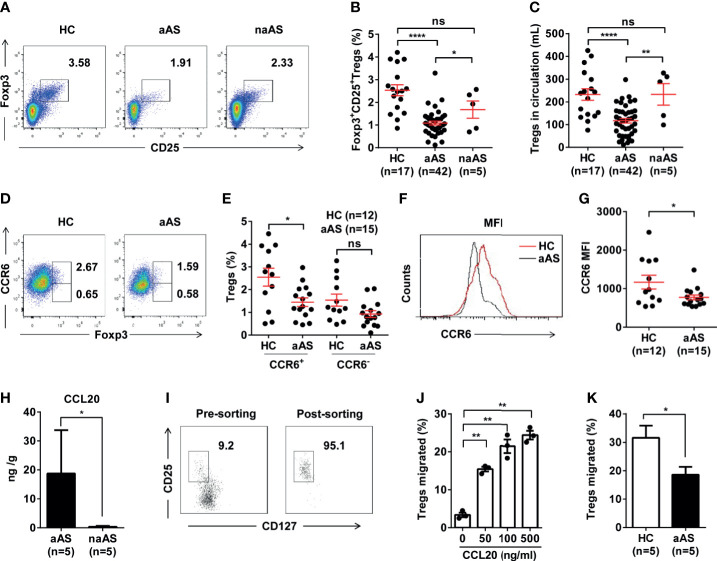
CCR6^+^CD25^+^Foxp3^+^Treg cell population is significantly decreased in peripheral blood of patients with allergic asthma. **(A)** Percentages of CD25^+^Foxp3^+^ Treg cells among peripheral CD3^+^CD8^-^T^-^cell subsets. Representative dot plots of HC, patients with allergic asthma (aAS), and patients with non-allergic asthma (naAS) are shown. **(B)** Frequencies and **(C)** absolute numbers of CD25^+^Foxp3^+^Treg cells in peripheral blood of HC (n = 17), patients with allergic asthma (n = 42) and patients with non-allergic asthma (n = 5). **(D)** Percentages of CCR6^+^Foxp3^+^Treg cells or CCR6^-^Foxp3^+^Treg cells among peripheral CD3^+^CD8^-^ T-cell subsets. Representative dot plots of HC, patients with allergic asthma (aAS). **(E)** Cumulative data of CCR6^+^Foxp3^+^Treg cells or CCR6^-^Foxp3^+^Treg cells in HC (n = 12) and aAS (n = 15). **(F)** CCR6 expression (MFI) of CD25^+^Foxp3^+^Treg subsets (gated on CD3^+^CD8^-^ T cells) of one healthy control and one patient with allergic asthma. One representative histogram overlay is shown. **(G)** Cumulative data of the MFI of CCR6 on CD25^+^Foxp3^+^Treg subsets. **(H)** CCL20 in induced sputum samples determined using ELISA. **(I)** CD3^+^CD4^+^CD25^+^CD127^-^Treg cells purified from the PBMCs of healthy individuals using flow cytometer sorting. The dot represents one data from an individual. **(J)** Cumulative data of the migration ratio of purified Treg cells cultured in the presence of CCL20 at different concentrations (0, 50, 100, 500 ng/mL). **(K)** Comparison of migration ratio of purified Treg cells between HC (n = 5) and aAS (n = 5). Bars represent the mean ± SEM. *P < 0.05, **P < 0.01, ****P < 0.0001. ns, not significant.

Compared with the HC (n = 17) and non-allergic asthma subjects (naAS, n = 5), the percentage and number of CD25^+^Foxp3^+^ Treg cells was markedly decreased in patients with allergic asthma (aAS, n = 42, **Figures 1A–C**). Treg cells were defined as described in the Materials and Methods section. Next, we compared the percentages of circulating CCR6^+^Foxp3^+^ Treg cells and CCR6^-^Foxp3^+^ Treg cells among peripheral CD3^+^CD8^-^ T cells between HC and aAS. In comparison with the HC (n = 12), the percentage of CCR6^+^Foxp3^+^ Treg cells was markedly decreased in aAS (n = 15). Meanwhile, there were no changes in the percentage of CCR6^-^Foxp3^+^ Treg cells (**Figures 1D, E**). **Figure 1F** shows that the mean fluorescence intensity (MFI) of CCR6 in CD25^+^Foxp3^+^ Treg cells of aAS is lower than that of HC. Furthermore, there was a marked decrease in CCR6 expression on CD25^+^Foxp3^+^ Treg cells in asthma patients as compared to the HC (**Figure 1G**). These observations suggest that the circulating CCR6^+^ Treg cells might have migrated to the airways of patients with allergic asthma. Subsequently, we collected induced sputum from five patients with aAS and five patients with naAS, and also detected CCL20, a unique ligand for CCR6. Notably, we found an increased level of CCL20 in the sputum of patients with aAS (**Figure 1H**). To identify the role of CCR6-CCL20 in Treg cells migration, a high proportion of Treg cells (> 95%) was sorted from HC (**Figure 1I** and **Supplementary Figure 2A**), and a transwell assay was used. **Figure 1J** shows that CCL20 promotes Treg cells migration in a dose-dependent manner. By comparing the differences in Treg cells migration, we found that Treg cells from HC had a higher migration ability than those from patients with aAS (**Figure 1K**). These results suggest that CCL20, which was markedly increased in the lungs of patients with aAS, might promote CCR6^+^ Treg cells migration. The reduced peripheral CCR6^+^ Treg cells might influence the development of allergic asthma.

### Circulating Foxp3^+^Treg Cell-Specific Expression of *CD4* and *CD25*

**Figure 2:**
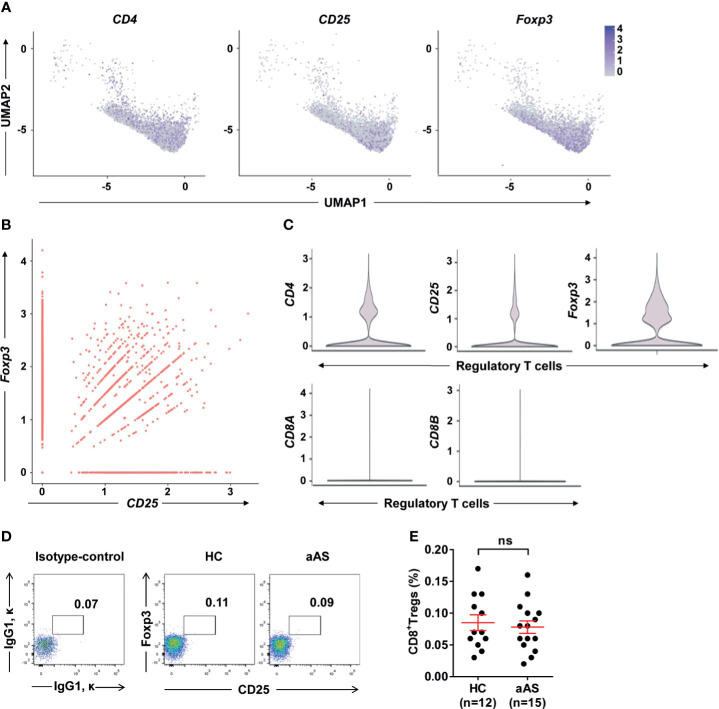
Single-cell gene expression in cluster of Foxp3^hi^ Treg cells. **(A)** UMAP plot depicts expression of surface markers transcripts, *CD4*, *CD25*, and *Foxp3* in resting Foxp3^+^ T cells cluster. **(B)** Feature scatter dot plot shows the correlation expression of *Foxp3* and *CD25*. **(C)** Violin plot shows the expression of *CD25, CD4, Foxp3, CD8A*, and *CD8B* in the cluster of regulatory T cells. **(D)** Percentages of CD25^+^Foxp3^+^Treg cells among peripheral CD3^+^CD8^+^T^-^cell subsets. **(E)** Cumulative data of CD8+ Treg cells in HC (n = 12) and aAS (n = 15). Representative dot plots of the healthy donor (n = 12) and patients with asthma (n =15) are shown. ns, not significant.

Treg cells expressing CD8 have been previously reported (30). These cells were observed in tonsils, but rarely detected in peripheral blood (31). These CD8^+^ Treg cells were also rarely reported in allergic asthma. To explore whether altered peripheral Treg cells originate mainly from CD4^+^ or CD8^+^ Treg cell subsets, we gated on Foxp3^+^CD25^+^ Treg cells and separated them into CD8^+^ and CD8^-^ Treg (mainly CD4^+^ Treg) cells (**Supplementary Figure 3A**). We found CD8^-^ Treg (mainly CD4^+^ Treg) cells were more dominant than CD8^+^ Treg cells. And no significant difference was observed between HC and aAS (**Supplementary Figure 3B**). Next, we used ScRNA-seq to capture the transcriptional landscape at the single-cell resolution. The samples of ScRNA-seq were obtained from two HC and two patients with aAS. The PBMCs of these subjects were collected, and the CD3^+^ T cells were then sorted and subjected to scRNA-seq using the 10 × platform. Then, we used Seurat to normalize and cluster the gene expression matrix and identified the cell subsets. The results were visualized using uniform manifold approximation and projection (UMAP) for dimension reduction. The three genes (*CD4, CD25*, and *Foxp3*) enriched in the cluster were used to assign cluster-specific cell identities (**Figure 2A**). Significantly more differentially expressed genes (DEGs) were shown in **Supplementary Figure 4**. Among them, *CD25* was highly co-expressed with *Foxp3* in nearly 50% of Foxp3^+^ Treg cells (**Figure 2B**). Moreover, the most prominent features were the high levels of *CD25, Foxp3*, and *CD4* expression, but not *CD8A* or *CD8B* (**Figure 2C**). Collectively, RNA-seq provided evidence of circulating Treg cell-specific expressed of *CD4, CD25*, and *Foxp3*, but not *CD8A/CD8B*. Subsequently, we explored whether a small amount of CD8^+^ Treg cells altered in aAS in comparison with HC. Our data showed that there was no significant difference in the percentage of CD8^+^CD25^+^Foxp3^+^ Treg cells between these two groups (**Figures 2D, E**).

### Inhibitory Function of Circulating Treg Cells Was Weakened in Patients With Allergic Asthma

**Figure 3:**
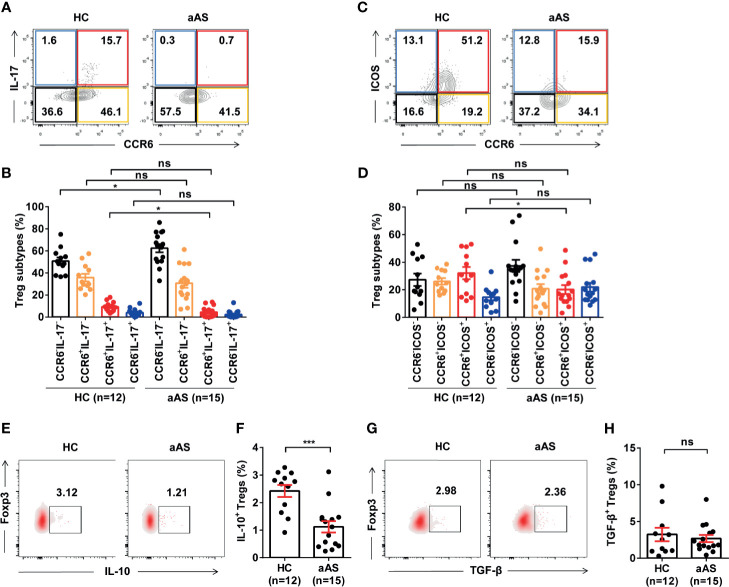
Peripheral Treg cell subsets and function are altered in patients with allergic asthma. **(A, B)** CCR6 *vs*. IL-17 expression of CD3^+^CD8^-^CD25^+^Foxp3^+^ Treg cells from the healthy donor (n =12) and patients with asthma (n = 15) analyzed with FACS. Representative dot plots are shown. **(C, D)** ICOS *vs*. CCR6 expression of CD3^+^CD8^-^CD25^+^Foxp3^+^ Treg cells from the healthy donor (n =12) and patients with asthma (n = 15) analyzed using FACS. Representative dot plots are shown. **(E, F)** IL-10^+^Foxp3^+^ Treg cells from the healthy donor (n = 12) and patients with asthma (n =15) analyzed with FACS. Representative dot plots are shown. **(G, H)** TGF-β^+^Foxp3^+^ Treg cells from the healthy donor (n = 12) and patients with asthma (n = 15) analyzed using FACS. Representative dot plots of are shown. Bars represent the mean ± SEM. *P < 0.05, ***P < 0.001; ns, not significant.

It has been reported that CCR6 induces the phosphorylation of Akt, mTOR, and STAT3 molecules, which are involved in suppressing *Foxp3* expression and promoting Th17 differentiation (32, 33). We measured the function of Treg cells in the enrolled subjects. First, we compared the co-expression of IL-17 *vs*. CCR6 (**Figure 3A**) and ICOS *vs* CCR6 (**Figure 3C**) on the CD25^+^Foxp3^+^ Treg cells (gated on CD3^+^CD8^-^ T cells) between HC (n =12) and patients with aAS (n = 15). Interestingly, we found that different Treg cells subtypes separate populations. The CCR6^+^IL-17^+^ Treg cells subsets (**Figure 3B**) and the CCR6^+^ICOS^+^ Treg cells subsets (**Figure 3D**) decreased significantly in patients with allergic asthma compared with HC. However, the CCR6^-^IL-17^-^ Treg cells subsets increased in patients with allergic asthma (**Figure 3D**).The percentage of Foxp3^+^IL-17A^+^ Treg cells was significantly lower in patients with aAS than in HC subjects (1.79% *vs*. 9.06%, median, respectively; *P* < 0.05, **Figure 3B**). Furthermore, patients with aAS displayed a lower percentage of Foxp3^+^ICOS^+^ Treg cells than HC subjects (15.9% *vs*. 30.5%, median, respectively; *P* < 0.05; **Figure 3D**). Consistently with previous studies (34), when comparing patients with aAS, Treg cells from HC subjects produced significant amounts of IL-10 (2.7% *vs*. 1.1%, median, respectively; *P* < 0.05; **Figures 3E, F**). No significant difference in TGF-β production was found between the two groups (2.5% *vs*. 1.8%, respectively; *P* > 0.05; **Figures 3G, H**). These results suggest that the Treg cells subsets are altered and inhibitory function of circulating Treg cells was weakened in patients with aAS.

### No correlation Between CCL20 and CCR6^+^Treg Cells in a Mouse Model Induced by OVA

**Figure 4:**
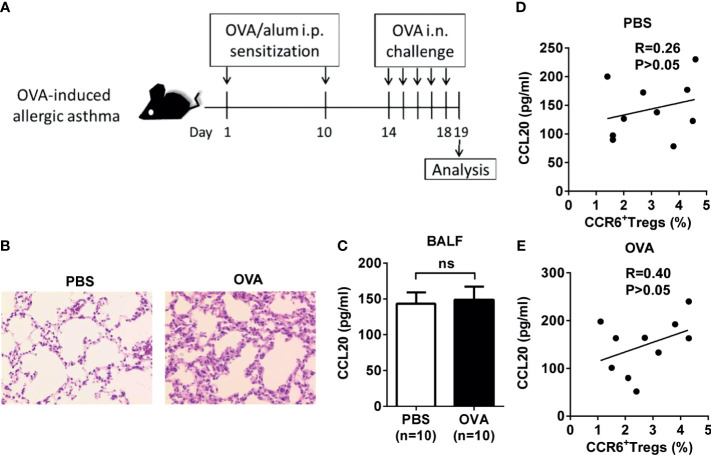
Association of CCL20 and CCR6^+^ Treg cells expression in OVA-induced allergic asthma mice. **(A)** Experimental scheme. Mice were sensitized and challenged as described (i.p., intraperitoneal, i.n., intranasal). **(B)** H&E staining of lung tissues. **(C)** Level of CCL20 in BALF analyzed using ELISA. PBS group (n = 10), OVA group (n = 10). **(D, E)** Relationship between percentage of CCR6^+^ Treg cells and CCL20 in controls (n = 10) and OVA-induced allergic asthma mice (n = 10). Spearman correlation coefficient (R) and p-values are indicated. ns, not significant.

To further elucidate changes in the phenotype and function of Treg cells in inflammatory lungs, we used the classic allergen-induced airway high reactivity (AHR) mouse models. First, an experimental model of OVA-induced AHR, airway eosinophilia, and type 2 cytokine production was used to monitor the activities of CCL20 and CCR6^+^ Treg cells (**Figure 4A, B**). Regardless of the treatment, the amount of CCL20 detected in the BALF was similar between the control and OVA-induced mice (**Figure 4C**). Moreover, the concentration of CCL20 protein in the lungs was not correlated with the frequency of CCR6^+^ Treg cells in the BALF (**Figures 4D, E**).

### Determination of IL-17^+^ Treg Cells in the Mouse Lung Induced by HDM

**Figure 5:**
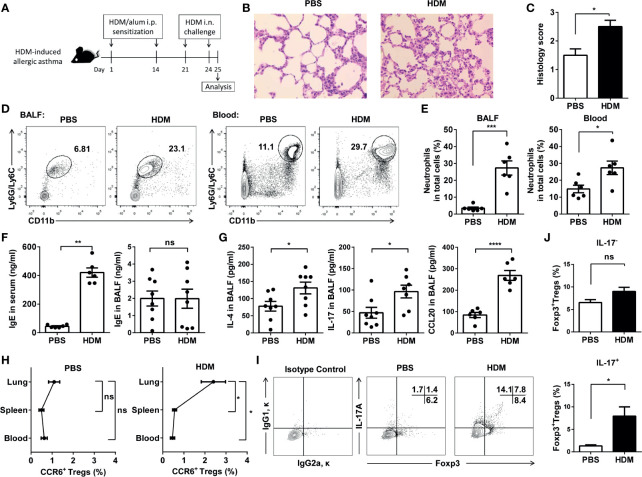
CCR6^+^Treg cells accumulated in lungs during HDM-induced allergic airway inflammation. **(A)** Experimental scheme. Mice were sensitized and challenged as described. **(B)** H&E staining of the lung tissues. **(C)** Determination of pathology score as described in Materials and Methods (n = 5 mice). **(D, E)** Neutrophils in the BALF and blood from asthmatic (n = 6) and control mice (n = 6) were analyzed by FACS. Neutrophils were identified as CD11b^+^Ly6G/Ly6C^+^ cells. **(F)** Determination of IgE secretion in serum and BALF using ELISA. **(G)** Determination of IL-4, IL-17 and CCL20 levels in BALF. **(H)** FACS analysis of CCR6^+^ Treg cells in the lung, spleen, and blood from HDM-induced asthmatic and control mice. Identification of CCR6^+^ Treg cells as CD4^+^CCR6^+^CD25^+^CD127^-^ cells. **(I, J)** FACS analysis of IL-17^-^Foxp3^+^ Treg cells and IL-17^+^Foxp3^+^ Treg cells in BALF from HDM-induced asthmatic (n = 5) and control mice (n = 5). Data are representative of three independent experiments with ≥ four mice per group. *P < 0.05, **P < 0.01, ***P < 0.001; ns, not significant.

We then used a mice model with HDM-induced asthma. Mice were sensitized with HDM in alum and intranasally challenged with HDM (**Figure 5A**). On Day 25, histological studies of the lungs showed that HDM exposure induced significantly higher peri-bronchial and peri-vascular leukocyte infiltrates in the lungs (**Figures 5B, C**). By detecting the CD11b^+^Ly6G/Ly6C^+^ neutrophils from BALF and blood (**Supplementary Figure 5**), we confirmed that HDM induced the neutrophilic inflammation in these mice (**Figures 5D, E**). Total IgE and cytokines from the BALF and serum were detected by ELISA. IgE in serum, but not in the BALF, increased significantly from the mice induced by HDM compared with the controls (**Figure 5F**). Moreover, HDM-sensitized mice primarily produced IL-4, IL-17, and CCL20 (**Figure 5G**). In agreement with these findings, after HDM sensitization, the mouse lungs had markedly more CCR6^+^ Treg cells than in PBS-injected control mice (**Figure 5H**). IL-17 producing Foxp3^+^ Treg (Th17 like Treg) cells were significantly elevated in the lungs of the HDM-induced asthma mice, whereas Treg cells did not express Th17-type cytokines not altered (**Figures 5I, J**). These data indicated that these Th17-like Treg cells might increase in neutrophilic inflammation. In contrast to the suppression of lung allergic responses, CCR6^+^ Treg cells enhanced this response. This was associated with increased levels of IgE in serum, IL-4, IL-17, and CCL20 in the BALF.

### Migration of CCR6^+^ Treg Cells to Inflamed Lungs Induced by HDM *In Vivo*

**Figure 6:**
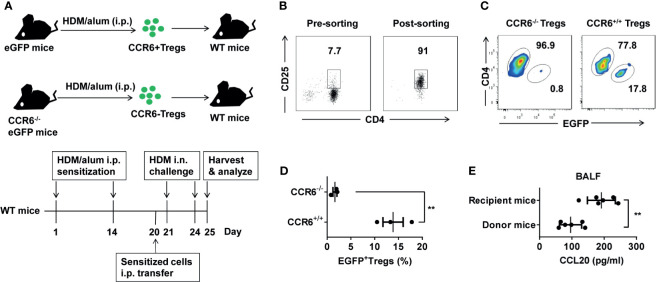
Recruited Treg cells could migrate to HDM-induced inflamed lungs *via* CCR6. **(A)** eGFP and CCR6^-/-^eGFP mice were treated with HDM/alum i.p. twice, and Treg cells were harvested. 1 × 10^5^ eGFP^+^ Treg cells were transferred i.p. into HDM-induced asthma recipient mice. HDM-sensitized recipient mice were challenged i.n. with HDM after the transfer for three days. **(B)** Detection of the purity of sorted CD3^+^CD4^+^CD25^+^ Treg cells population using FACS. **(C, D)** FACS analysis of the frequency of eGFP^+^ Treg cells (gated on CD3^+^CD4^+^ cells) in total CD4^+^ T cells from the lungs. **(E)** ELISA analysis of CCL20 in the BALF of the donor (n = 6) and recipient mice (n = 6) after transfer of eGFP^+^ Treg cells on Day 20. Data are representative of two independent experiments with three mice per group. Data are represented as the mean ± SEM. **P < 0.01.

**Figure 7:**
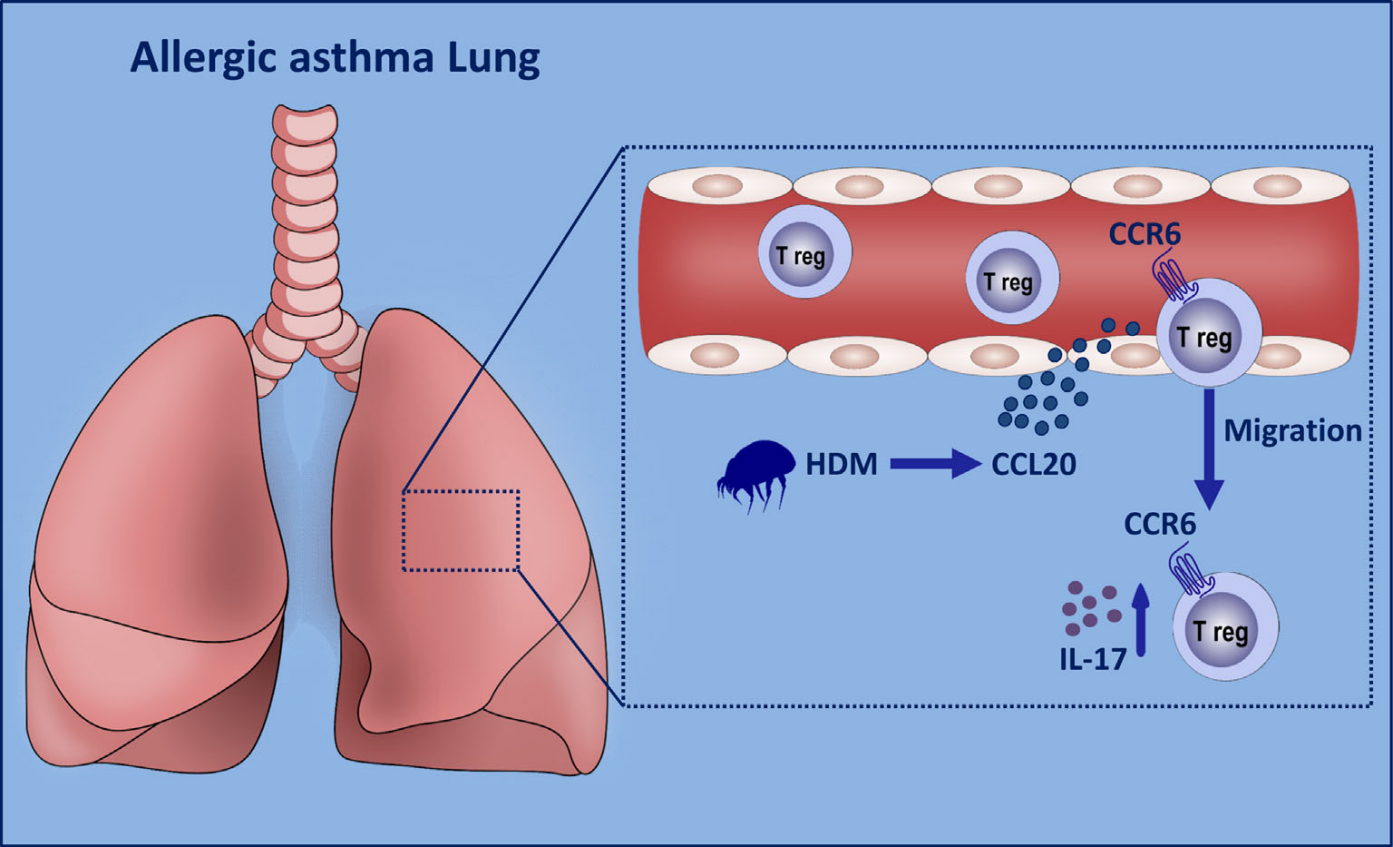
Graphical summary of proposed mechanism. When the lung epithelial cells are exposed to HDM stimulation, excessive amounts of CCL20 are released into the peripheral blood. CCR6^+^ Th17-like Treg cells tend to migrate to the inflamed lungs. These Tr17 cells release excessive amounts of IL-17 to aggravate asthma progression.

To determine whether CCL20 is a chemoattractant for CCR6^+^ Treg cells *in vivo*, we crossed eGFP transgenic mice expressing the GFP protein with CCR6^-/-^ mice; GFP marked cells that did not express CCR6 (**Figure 6A**). We separately sorted high proportion CD4^+^CD25^+^ Treg cells from these donors (**Figure 6B** and **Supplementary Figure 2B**) and adoptively transferred GFP^+^CCR6^-/-^ or GFP^+^CCR6^+/+^ donor derived CD4^+^CD25^+^ T cells into immunized WT mice, which were analyzed three days later (**Figure 6A**). In this migration assay, CCR6^+/+^ Treg cells showed a higher ability to migrate to the inflamed lungs than CCR6^-/-^ Treg cells (**Figures 6C, D**). Finally, we detected the CCL20 in BALF after the eGFP^+^ Treg cells were transferred on Day 25. These data confirmed that CCL20 in the lungs of recipient mice was higher after the Treg cells transfer (**Figure 6E**). Collectively, these findings suggest that CCR6^+^ Treg cells trafficking *via* CCL20, may be associated with the pathology of asthma (**Figure 7**).

## Discussion

In this study, we demonstrated that the pathogenesis of allergic asthma may be associated with CCR6^+^ Treg cells recruitment. Human allergic asthma constitutes an inflammatory microenvironment enriched with chemokines, such as CCL20, which promotes the migration of circulating CCR6^+^ Treg cells. We found that in the HDM-induced mouse model, there was a correlation between increased expression of inflammatory cytokines (such as IL-4, IL-17, and serum IgE) and Th17 like Treg cells. Nathan et al. found that stimulation of the respiratory epithelium by HDM-induced rapid secretion of CCL20, a chemokine central to the recruitment of immature DCs to the lungs (35). The effect is specific to HDM, because other aeroallergens, such as ragweed and cockroach, fail to elicit this response that is dependent on β-glucan structures in the HDM extract (35). HDM stimulates the innate immune system through the epithelial secretion of CCL20 to attract a specific subset of Treg cells to the lungs. Thus, the final profile of subsets of Treg cells in the lungs might be associated with allergic asthma responses.

Atopy is also considered to be the chief risk factor for the development of allergic disease. Here, we showed that a lower proportion of circulating Treg cells in peripheral blood from the patients with allergic asthma compared to non-allergic asthma. Consistently with our analyses of the induced sputum, only patients with allergic asthma showed higher levels of CCL20 expression. Allergens are associated with the development of allergic asthma (36), and the heterogeneity of circulating Treg cells lacks detailed explanation. The characterization of various subsets of Treg cells in asthmatic patients and comparison of their properties to those in HC is necessary. In the present study, we had characterized their phenotype based on the expression of CCR6, IL-17, and ICOS. As expected, the proportions of circulating Treg subsets varied between subjects with allergic asthma and HC. Previous studies showed that ICOS-mediated signaling was essential for the induction of Th2 cytokines (37). We found the proportions of CCR6^+^IL-17^+^ and CCR6^+^ICOS^+^ Treg cells were both lower in patients with allergic asthma than in healthy subjects. This relative defect of circulating Treg subsets in patients with allergic asthma could result from the recruitment by CCL20 derived from inflammatory lungs.

In fact, the proportion of Treg cells is significantly elevated in the BALF of patients with moderate to severe asthma as compared to that of healthy subjects (38). Therefore, we hypothesized that CCL20 may favor the recruitment and retention of Treg cells that express CCR6. Notably, most Treg cells in the lungs express CCR6 and IL-17 in the HDM-induced mouse model and exhibit an activated effector phenotype. CCL20 inhibits the differentiation of TGF-β-induced Treg (iTreg) and directs them toward the pathogenic Th17-lineage in a CCR6-dependent manner during gut inflammation (15). CCL20 downregulates FasL expression in differentiated iTreg cells *in vitro (* 15). Downregulation of regulatory molecules, such as CD39, CD73, and FasL, leads to impaired *in vitro* suppressive capacity of iTreg cells differentiated in the presence of CCL20 (32, 33). Moreover, Stat3 involvement in suppressing Foxp3 expression and promoting Th17 differentiation was also reported (39). These findings suggest that CCR6^+^ Treg cells marks Th17-like phenotypes have the potential to promote inflammation.

Several DEGs with reported Treg-related functions were observed in this study, including *TIGIT*, *CTLA4* in the cluster of *Foxp3* ^hi^ *IL2RA* ^hi^ T cells (**Supplementary Figure 4A**). We also explored the transcriptomic differences between CCR6^hi^ and CCR6^low^ Treg cells subsets. We found that 27 genes were upregulated more than two-fold in CCR6^hi^ cells compared to CCR6^low^ cells, and 13 were downregulated by the same factor (FC < –2) (**Supplementary Figure 4B**). Moreover, GO analysis indicates the enrichment of genes with annotations related to the inflammatory response, cytokine production, neutrophil degranulation, and Th17 cell differentiation in CCR6^hi^ Treg cells compared to CCR6^low^ Treg cells (**Supplementary Figure 4C**). The underlying mechanisms of CCR6^+^ Tregs less capable of suppression requires further investigation. Moreover, the relative contribution of CCR6^+^ Treg cells to the pathogenesis of allergic asthma is likewise unclear. Further studies must focus on the protective or detrimental roles of CCR6^+^ Treg cells.

In OVA-induced allergic asthma, the recruited inflammatory monocytes might promote inflammation by enhancing the Th2 response (29). In the current study, we did not observe any difference of CCL20 expression between OVA-induced allergic asthma and control mice. However, the numbers of CCR6^+^ Tregs was increased in OVA-induced models (**Supplementary Figure 6**). This indicated that other mediators were at play, in a non-CCL20 dependent manner. In HDM-induced allergic asthma, the Th17-derived cytokines are important in the induction and activation of neutrophils (25). Further, neutrophils can compromise the epithelial cell barrier function through secretion of proinflammatory stimuli (40). We also observed a significant increase in the frequencies of neutrophils in HDM-induced allergic asthma models. These mice were characterized by neutrophilia with a Th17-like CCR6^+^ Treg cells skew in asthmatic lungs and hyper-IgE levels in serum. We also found that most of the adoptively transferred CCR6^+/+^ Treg cells migrated to the lungs with higher levels of CCL20. These results indicate that CCR6 expression in Treg cells was necessary for the CCL20-dependent lung homing of these Treg cells. However, due to the limited data we obtained from OVA-induced allergic asthma models, the comprehensive comparison of these two models is difficult at this time. More detailed analysis of the OVA-induced allergic asthma models is required. Moreover, performing Treg cells-specific deletion of CCR6 will more precisely unravel the exact contribution of these cells to pulmonary inflammation in the future.

The Th17 pathway has been reported as a potential therapeutic target for steroid-insensitive asthma (41). IL-17 can induce the production of IL-8, and increased IL-17 levels have been reported in the sputum of patients with severe asthma (42). Moreover, T-cell plasticity also affects Treg cells and their functional properties in a mouse model (43, 44). Host-derived IL-6 is essential for the pathogenic conversion of iTreg cells to IL-17 production in the airways of recipient mice (45). The human thymus does not contain IL-17–producing Treg cells, suggesting that IL-17^+^Foxp3^+^ Treg cells are generated in the periphery (10). Massoud et al. reported that the IL-4RαR576 mutant enables the recruitment of the adaptor GRB2 to promote airway inflammation by a mechanism involving IL-4-directed iTreg cell differentiation toward the Th17 cell lineage (46). Recently, Harb et al. had identified Notch4 expressing Treg cells in response to ultrafine particles (47). This specific Treg population cannot properly suppress inflammation, because it produces Th17 and Th2 effector cytokines instead of immunoregulatory cytokines. In summary, these studies suggest that the acquisition of Treg cells in a Th17/Th2 cell–like program might play a key role in the pathogenicity of airway inflammation.

We suggest that Treg cells migrate to the airways *via* CCR6, but are incapable of attenuating allergic inflammation. CCR6^+^ Treg cells are the major proinflammatory Treg population that responds to HDM in human asthma exacerbations. Thus, blocking the CCR6-CCL20 pathway may prevent the conversion of HDM-specific Treg cells into Th17-like cells.

## Data Availability Statement

The single-cell RNA-seq data presented in the study are deposited in the Genome Sequence Archive in National Genomics Data Center, China National Center for Bioinformation, Chinese Academy of Sciences, under accession number HRA001164 that are publicly accessible at https://ngdc.cncb.ac.cn/gsa-human. The datasets presented in the study are deposited in online repositories. The accession link can be found below: https://www.jianguoyun.com/p/DcOWnHwQm_LACRjYzIYE. Further inquiries can be directed to the corresponding author.

## Ethics Statement

The studies involving human participants were reviewed and approved by the ethics committee of the First Affiliated Hospital of Jinzhou Medical University. The patients/participants provided their written informed consent to participate in this study. The animal study was reviewed and approved by The Committee on Animal Experimentation of Jinzhou Medical University.

## Author Contributions

XS and HZ performed most of the experiments and wrote a large part of the manuscript. SL carried out the mouse sensitization and challenge tests, ELISA, and generated part of the data and wrote part of the manuscript. ZW and YZ helped conduct the experiments. HX, LC, and RC performed the clinical studies. JZ performed the single-cell RNA-seq data analysis. SH designed and organized the study and wrote the final draft of the manuscript. All authors contributed to the article and approved the submitted version.

## Funding

This project was sponsored by grants from the National Natural Science Foundation of China (31800761, 81601372), the Natural Science Foundation of Liaoning Province (20170520057, 201601356), the Educational Department of Liaoning Province (JYTQN201918), the Liaoning Revitalization Talents Program (XLYC2007097), the Leader Team Project of Jinzhou Medical University (2017-2020), and grants from the Climbing Scholar Project in Liaoning province (2018-38).

## Conflict of Interest

The authors declare that the research was conducted in the absence of any commercial or financial relationships that could be construed as a potential conflict of interest.

## Publisher’s Note

All claims expressed in this article are solely those of the authors and do not necessarily represent those of their affiliated organizations, or those of the publisher, the editors and the reviewers. Any product that may be evaluated in this article, or claim that may be made by its manufacturer, is not guaranteed or endorsed by the publisher.

## Abbreviations

HDM, house dust mite; OVA, ovalbumin; PBMCs, peripheral blood mononuclear cells; BALF, bronchoalveolar lavage fluid; FACS, fluorescence activated cell sorting.
