# Supplementary material for: Reduced CCR6+IL-17A+Treg Cells in Blood and CCR6-Dependent Accumulation of IL-17A+Treg Cells in Lungs of Patients With Allergic Asthma
Source: Front Immunol. 2021 Aug 23;12:710750. doi: 10.3389/fimmu.2021.710750 (PMC8419235; doi:10.3389/fimmu.2021.710750)
Supplement: Supplementary file 1 [file DataSheet_1.docx]

**Supplementary Materials and Methods**

**Single-cell RNA-seq**

A Ficoll-Paque density gradient for separation of human PBMCs. Next, CD3^+^ T cells were sorted according to the official recommendations from 10× Genomics for scRNA-seq analysis. The sorted CD3^+^ T cells were then counted and resuspended at a concentration of 1000 cells/μL, aiming for an estimated 8000 cells per library, following the instructions of single-cell 3ʹ solution v2 reagent kit (10× Genomics). Briefly, the cell suspensions were loaded onto a chromium single-cell chip along with reverse transcription master mix and 3ʹ gel beads. After the generation of single-cell gel bead-in-emulsions (GEMs), reverse transcription was performed using a C1000 TouchTM Thermal Cycler (Bio-Rad). The amplified cDNA molecules were then purified with SPRIselect beads (Beckman Coulter). Single-cell libraries were then constructed following fragmentation, end repair, polyA-tailing, adaptor ligation, and size selection according to the manufacturer’s standard protocols. Each sequencing library was generated with a unique sample index. Libraries were sequenced on the Illumina HiSeq X Ten platform.

**ScRNA-seq data processing**

The raw sequencing data of patients with allergic asthma and healthy controls were processed using Cell Ranger (version 3.1.0). To display the cells in a two-dimensional space, we performed principal component analysis on the integrated dataset and used the first 50 principal components (PCs) for uniform manifold approximation and projection (UMAP) analysis.

**Supplementary Figures**


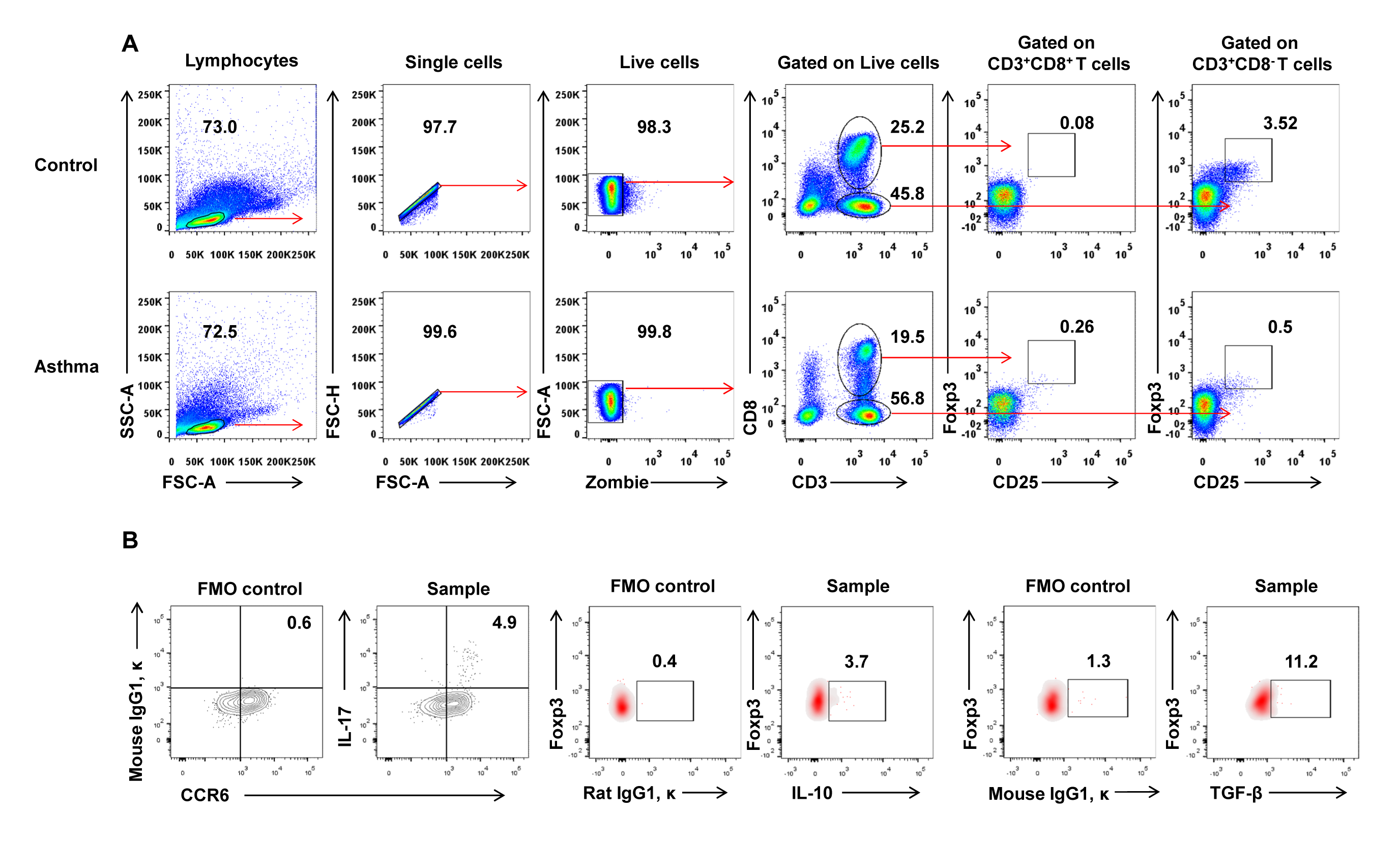


**Supplementary Figure 1. The** **flow cytometry gating strategy for human Treg cells.** (A) Lymphocytes were identified by forward and side scatter (FSC-A vs. SSC-A), and single cells were identified (FSC-A vs. FSC-H). Next, dead cells (Zombie) were excluded, and CD8^-^ or CD8^+^ Treg cells were defined as CD3^+^CD8^-^CD25^+^Foxp3^+^ or CD3^+^CD8^+^CD25^+^Foxp3^+^. (B) The fluorescence minus one controls (FMOs) were used to identify IL-17, IL-10 and TGF-β.

**Supplementary Figure 2.** **The** **flow cytometry gating strategy for the sorting of Treg cells.** (A) Human Treg cells are gated as CD3^+^CD4^+^CD25^+^CD127^-^ within the lymphocytes gate which were excluded cell debris (FSC-A vs. SSC-A), doublets (FSC-A vs. FSC-H) and dead cells (Zombie positive). (B) Mice Treg cells are gated as CD4^+^CD25^+^ within the lymphocytes gate which were excluded cell debris (FSC-A vs. SSC-A), doublets (FSC-A vs. FSC-H) and dead cells (Zombie positive).


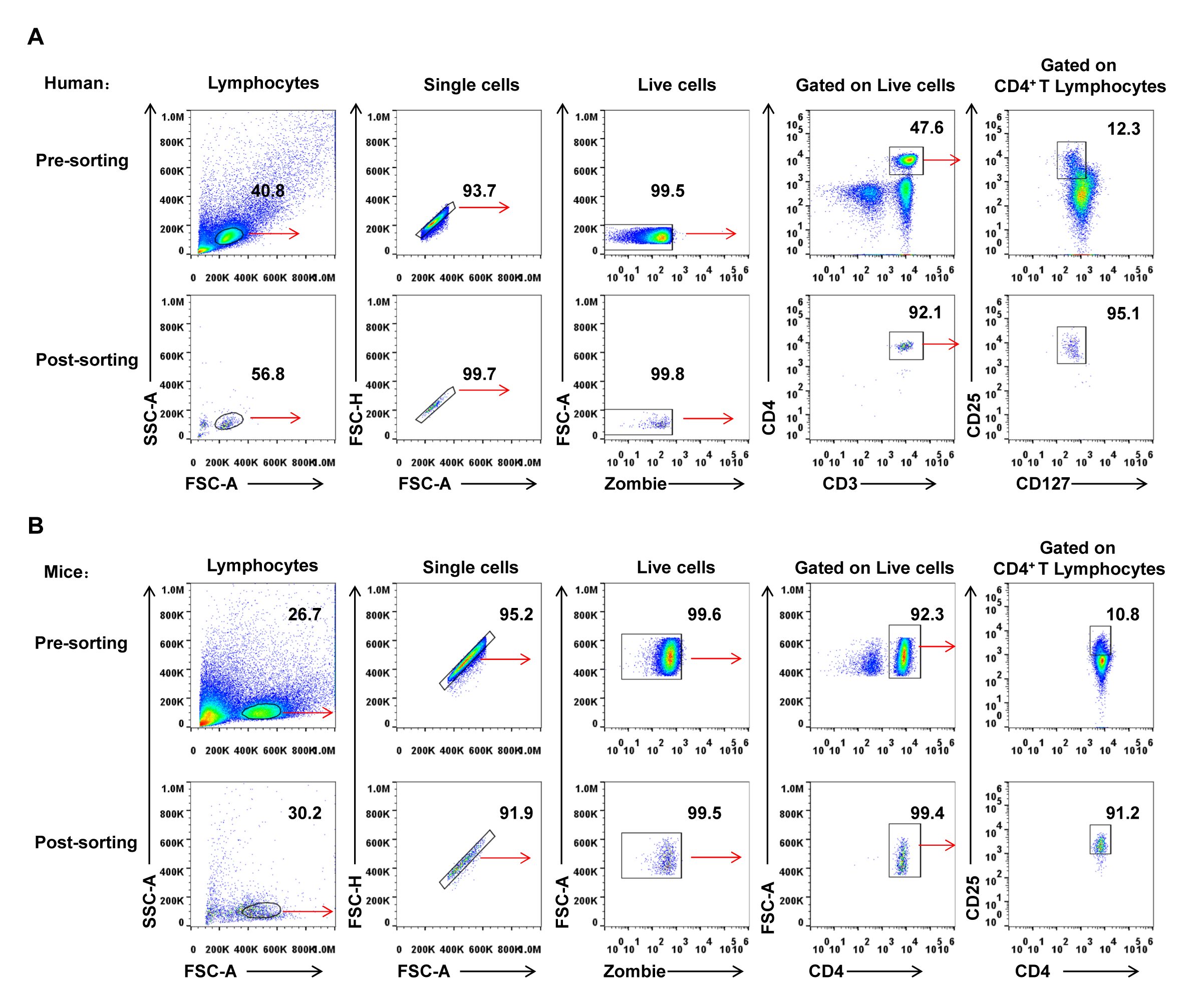


**
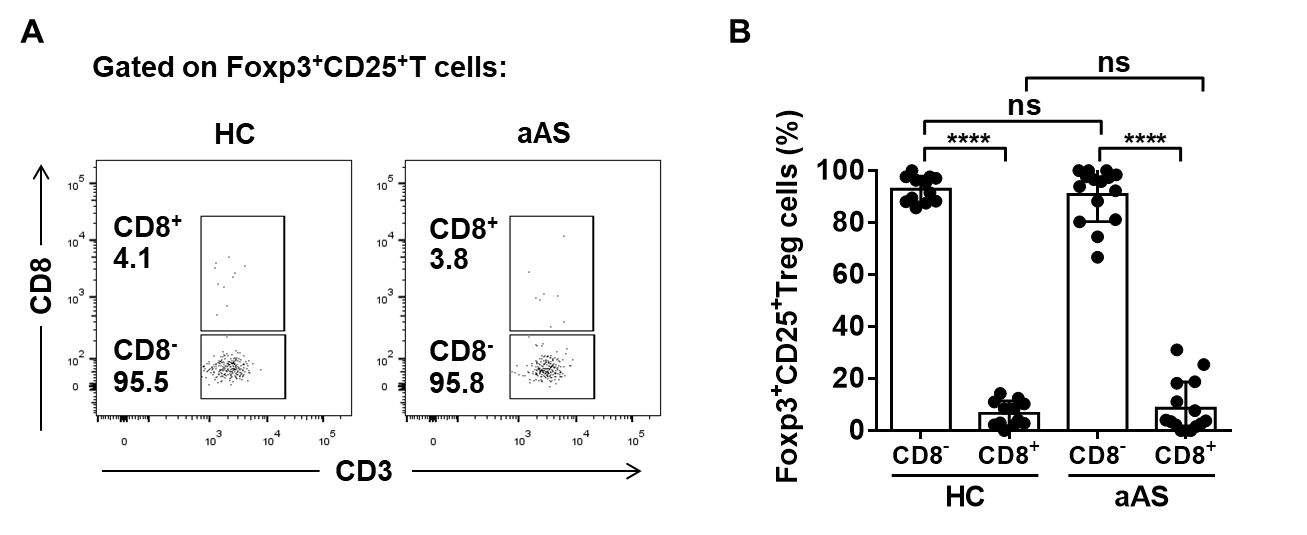
**

**Supplementary Figure 3. Foxp3^+^CD25^+^ T cells (CD3^+^CD8^+^ or CD3^+^CD8^-^ T cells) from HC and aAS were analyzed using flow cytometry.** A. Foxp3^+^CD25^+^ T cells were separated them into CD8^+^ T cells and CD8^-^ T cells (mainly CD4^+^ T cells). B. Foxp3^+^CD25^+^ Treg cells originate mainly from CD8^-^ Treg cell subsets (mainly CD4^+^ T cells). No significant difference was observed between HC and aAS.


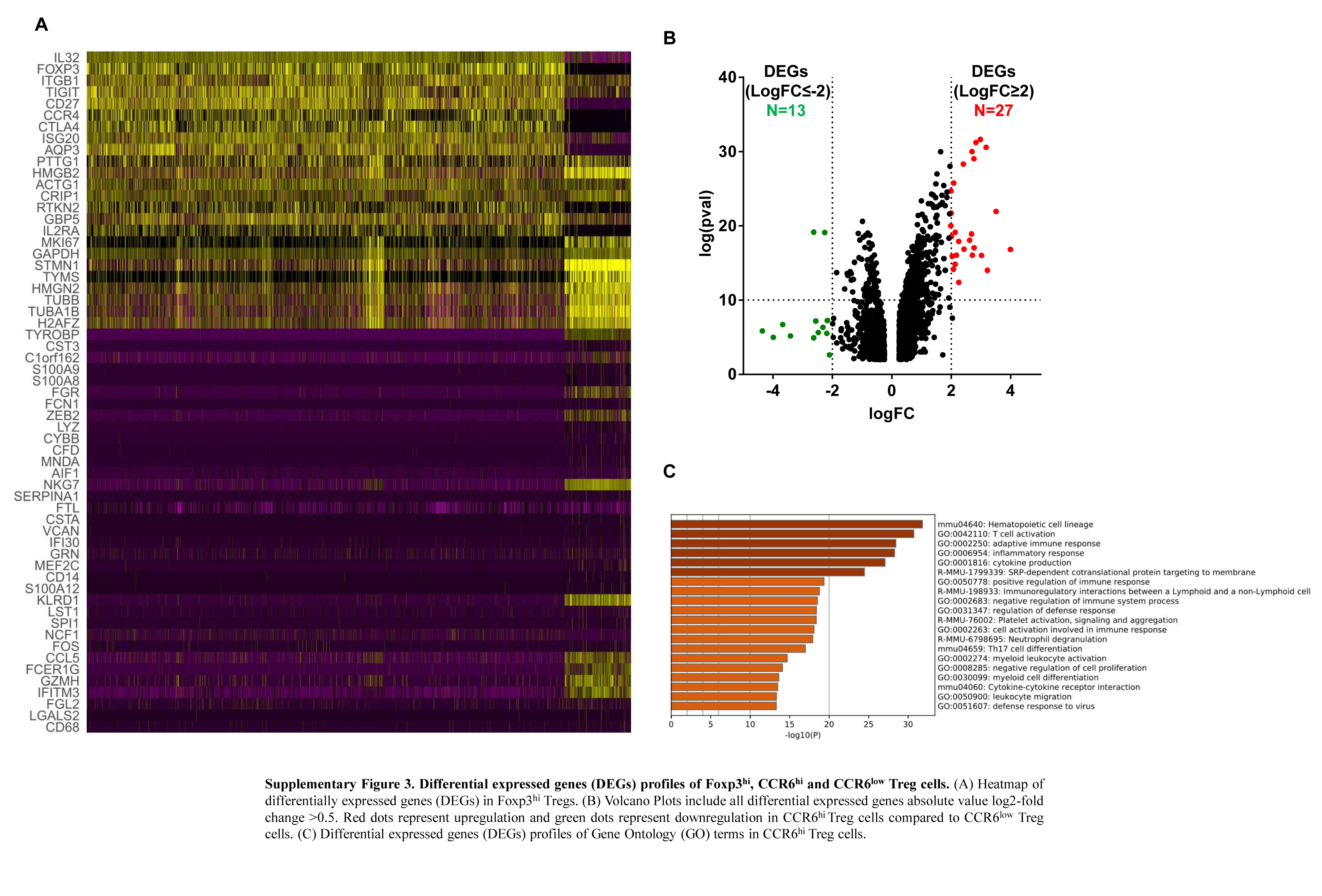


**Supplementary Figure 4. Differential expressed genes (DEGs) profiles of Foxp3^hi^, CCR6^hi^ and CCR6^low^ Treg cells.** (A) Heatmap of differentially expressed genes (DEGs) in Foxp3^hi^ Tregs. (B) Volcano Plots include all differential expressed genes absolute value log2-fold change >0.5. Red dots represent upregulation and green dots represent downregulation in CCR6^hi^ Treg cells compared to CCR6^low^ Treg cells. (C) Differential expressed genes (DEGs) profiles of Gene Ontology (GO) terms in CCR6^hi^ Treg cells.


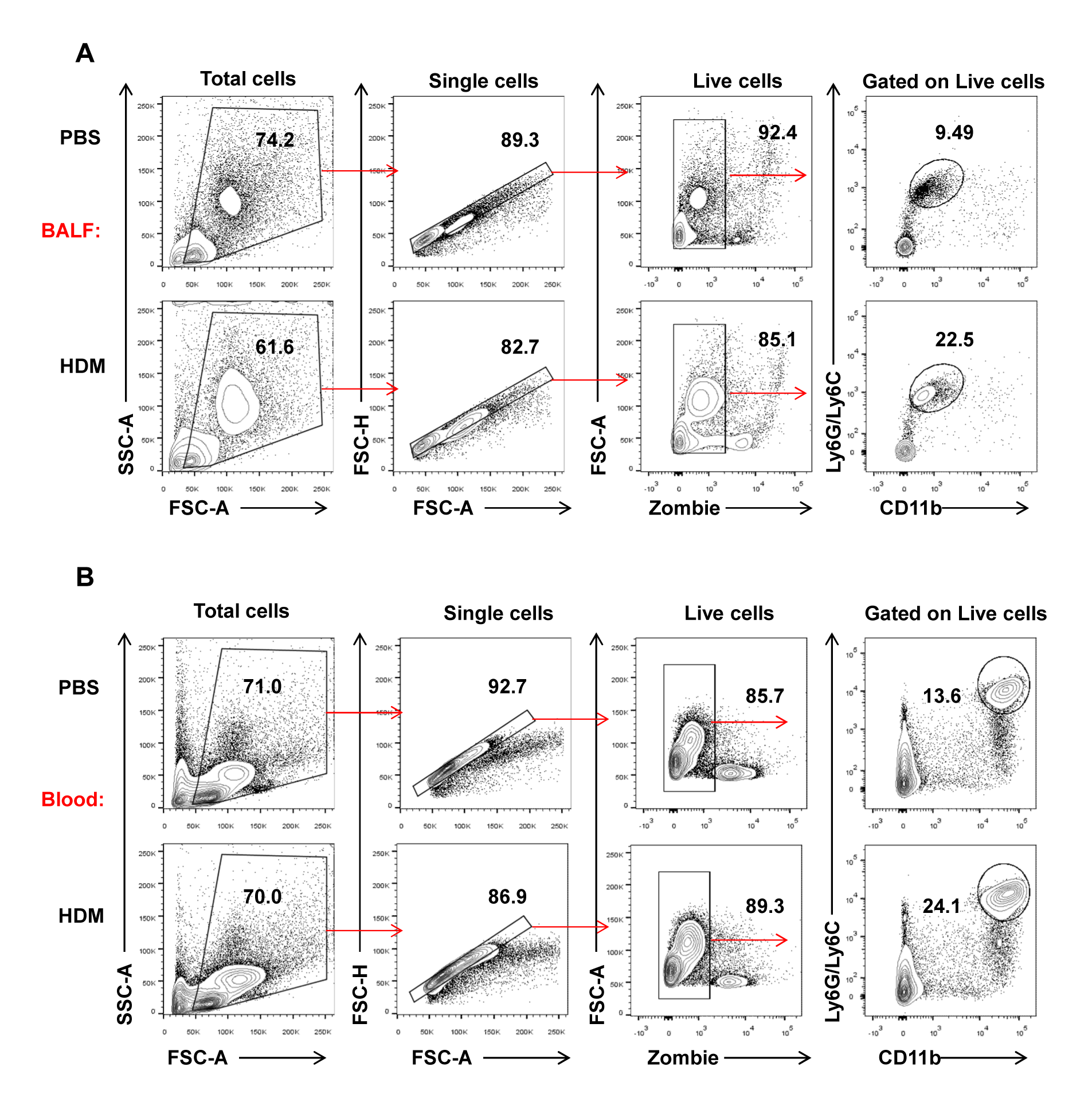


**Supplementary Figure 5. The** **flow cytometry gating strategy for neutrophils in BALF and blood of mice.** (A) Total cells were identified by forward and side scatter (FSC-A vs. SSC-A), and single cells were identified (FSC-A vs. FSC-H). Next, dead cells (Zombie) were excluded, and Neutrophil were identified as CD11b^+^Ly6G/Ly6C^+^ cells.

**Supplementary Figure 6.** The absolute numbers of CCR6^+^ Treg cells of the lungs in controls (n = 6) and OVA-induced allergic asthma mice (n = 6).
